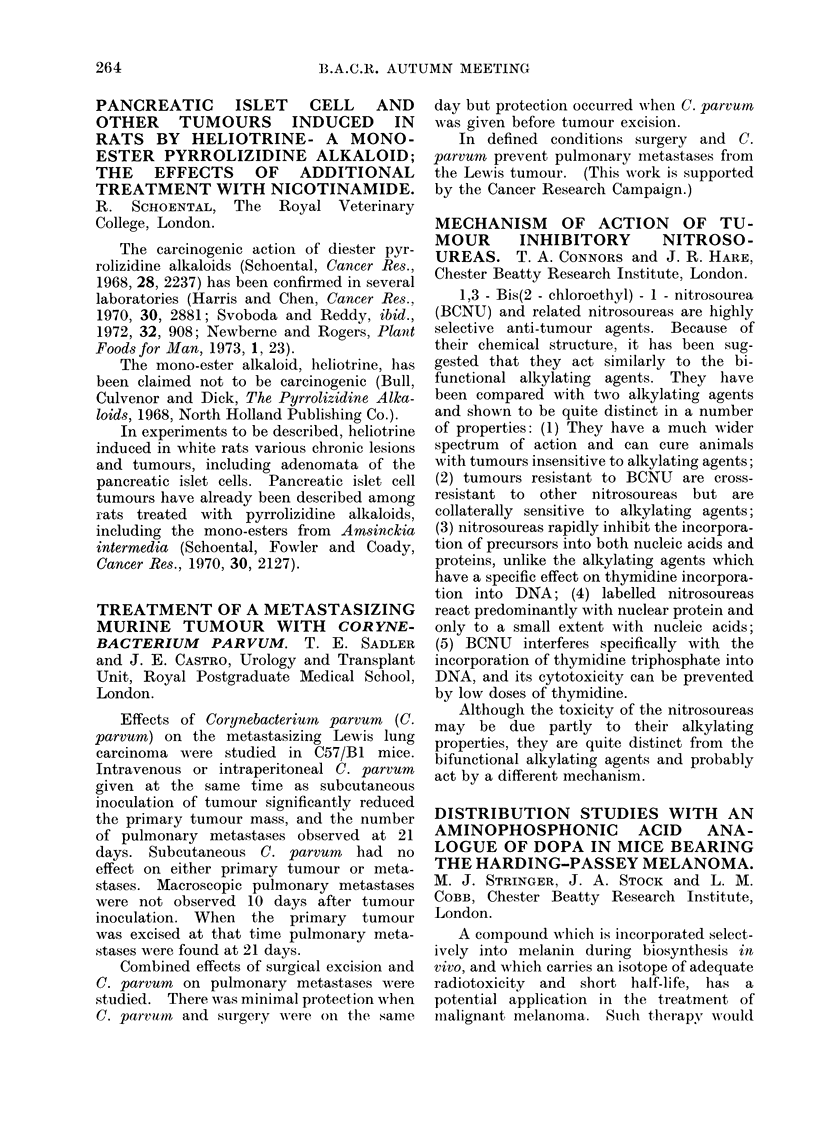# Proceedings: Treatment of a metastasizing murine tumour with Corynebacterium parvum.

**DOI:** 10.1038/bjc.1975.50

**Published:** 1975-02

**Authors:** T. E. Sadler, J. E. Castro


**TREATMENT OF A METASTASIZING MURINE TUMOUR WITH *CORYNEBACTERIUM PARVUM*.** T. E. SADLER and J. E. CASTRO, Urology and Transplant Unit, Royal Postgraduate Medical School, London.

Effects of *Corynebacterium parvum* (*C. parvum*) on the metastasizing Lewis lung carcinoma were studied in C57/B1 mice. Intravenous or intraperitoneal *C. parvum* given at the same time as subcutaneous inoculation of tumour significantly reduced the primary tumour mass, and the number of pulmonary metastases observed at 21 days. Subcutaneous *C. parvum* had no effect on either primary tumour or metastases. Macroscopic pulmonary metastases were not observed 10 days after tumour inoculation. When the primary tumour was excised at that time pulmonary metastases were found at 21 days.

Combined effects of surgical excision and *C. parvum* on pulmonary metastases were studied. There was minimal protection when *C. parvum* and surgery were on the same day but protection occurred when *C. parvum* was given before tumour excision.

In defined conditions surgery and *C. parvum* prevent pulmonary metastases from the Lewis tumour. (This work is supported by the Cancer Research Campaign.)